# Supplementary material for: NCOA7 Regulates Growth and Metastasis of Clear Cell Renal Cell Carcinoma via MAPK/ERK Signaling Pathway
Source: Int J Mol Sci. 2023 Jul 18;24(14):11584. doi: 10.3390/ijms241411584 (PMC10380801; doi:10.3390/ijms241411584)
Supplement: Supplementary file 1 [file ijms-24-11584-s001.zip › ijms-2467188-supplementary.pdf]

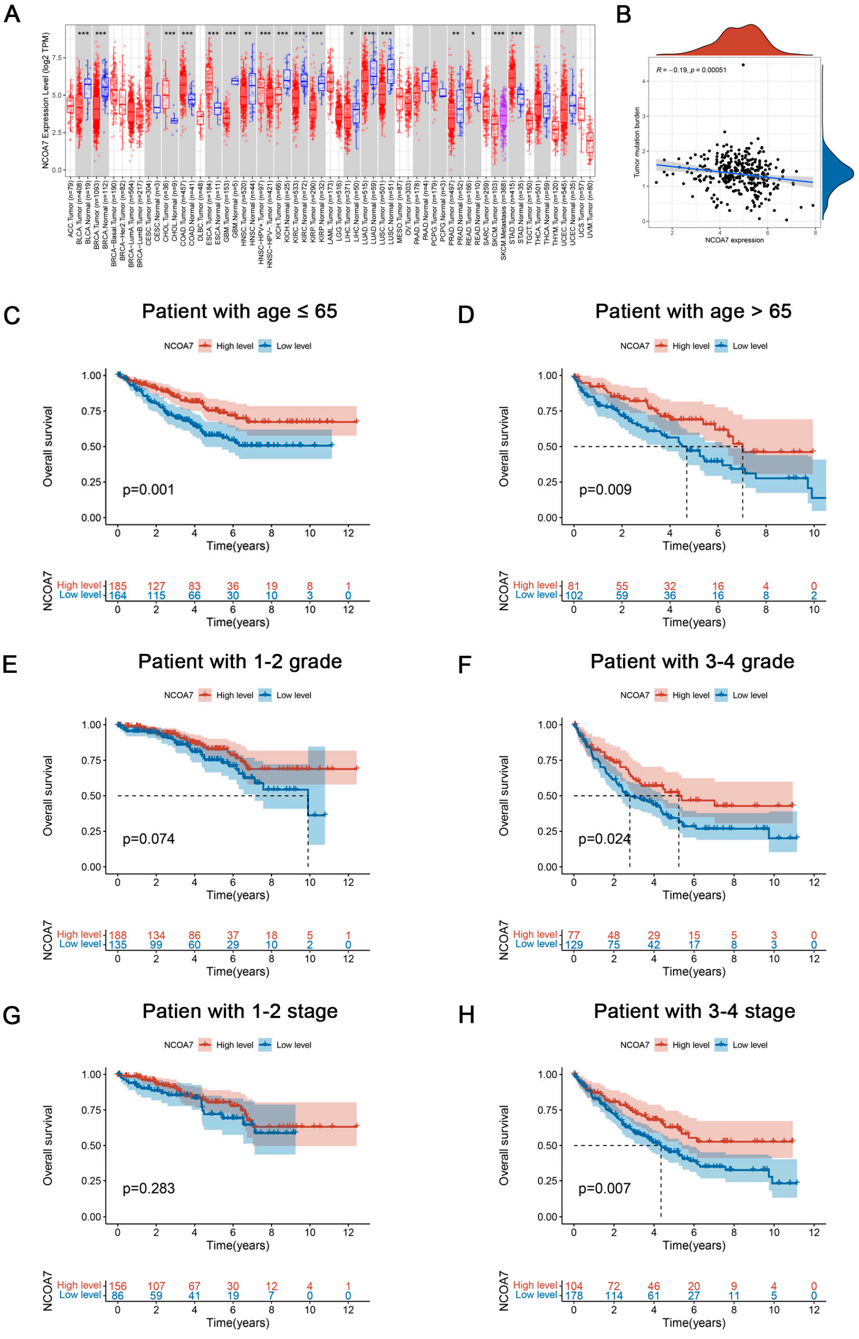

Figure S1. Pan-carcinoma expression of NCOA7, and the effect of NCOA7 on the survival of ccRCC patients with different ages, grades, and stages. (**A**) The expression profile of NCOA7 across all tumor samples and normal tissues. (**B**) Correlation of NCOA7 expression with tumor mutational burden. (C-H) Overall survival curve of ccRCC patients with low and high NCOA7 expression at ≤65 years (**C**), > 65 years (**D**), 1–2 grade (**E**), 3–4 grade (**F**), 1–2 stage (**G**), and 3–4 (**H**), respectively. * < 0.05, ** $p < 0.01$, *** $p < 0.001$.
